# Unveiling the Potential of Redox Chemistry to Form Size-Tunable, High-Index Silicon Particles

**DOI:** 10.1021/acs.chemmater.4c01439

**Published:** 2024-08-28

**Authors:** Megan A. Parker, Safa Khaddad, Nicolas Fares, Anissa Ghoridi, David Portehault, Sébastien Bonhommeau, Yacine Amarouchene, Patrick Rosa, Mathieu Gonidec, Glenna L. Drisko

**Affiliations:** † University of Bordeaux, CNRS, Bordeaux-INP, ICMCB, UMR 5026, F-33600 Pessac, France; ‡ University of Bordeaux, CNRS, LOMA, UMR 5798, F-33405 Talence, France; § Laboratoire de Chimie de la Matière Condensée de Paris (LCMCP), Sorbonne Université CNRS, F-75005 Paris, France; ∥ University of Bordeaux, CNRS, Bordeaux-INP, ISM, UMR 5255, F-33400 Talence, France

## Abstract

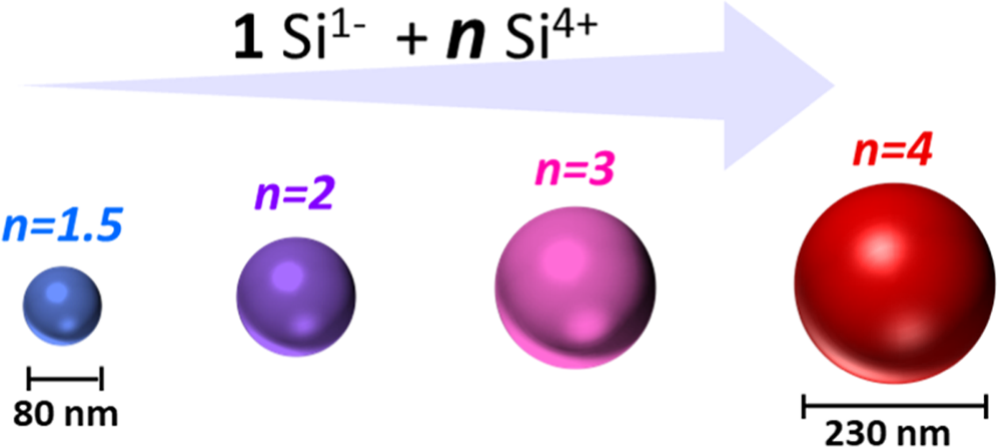

Silicon particles of intermediate sizes (75–200 nm) scatter visible wavelengths, making them promising candidates for optical devices. The solution synthesis of silicon particles in this size range, however, has proved challenging for chemists over the past few decades. Here, a solution-phase synthesis provides a pathway toward reaching size tunability between 45 and 230 nm via changing the reactant ratio in the reaction between a silicon Zintl phase (Na_4_Si_4_) with an amidinate-stabilized Si(IV) coordination complex. Coherent domain sizes, determined from powder X-ray diffraction, show that the crystallite sizes are uniform across all particle sizes, perhaps indicating an aggregation mechanism for particle growth. The amidinate ligands act to stabilize the particle surface. Combined surface techniques (ToF-SIMS, FTIR, and X-ray photoelectron spectroscopy) confirm the presence of amidinate ligands, as well as primary amine and a passive oxidation layer on the surface of the particles. The refractive index is measured for an individual particle using holographic optical microscopy, displaying a refractive index of nearly 4.1 at a wavelength of 532 nm. Thus, these particles should scatter light intensely at visible wavelengths, making them promising candidates for optical manipulation.

## Introduction

Crystalline, nonporous Si particles of certain diameters have extraordinary optical properties thanks to the polarization oscillations within individual atoms known as Mie resonances, contributing to intense light scattering. Silicon particles of appropriate size can generate electric field enhancement, directional scattering, or structural coloration. The Purcell effect can also yield the spontaneous emission of nearby emitters when in close contact with Si structures.^1−3^ When assembled, these particles have potential in the development of miniaturized, flat optical components.^4,5^ Although, the perspectives for these objects are expansive, few synthesis methods are able to produce the large quantities of Si particles needed for the fabrication of optical devices, particularly with the required crystallinity and size- and shape control.

Moreover, a range of sizes is needed to cover the diversity of optoelectronic applications. The size of silicon particles is limited to a few nanometers for most solution routes.^6−8^ Decomposition under supercritical conditions produces particles that are amorphous and porous,^9^ yielding weak Mie resonances outside of the visible region, except in the case of co-condensation with a second precursor.^200^ Disproportionation of silicon-rich oxides produces highly optically responsive particles, but yield is low, particularly after size-separation, and the synthesis requires the use of hazardous hydrofluoric acid to liberate the silicon particles.^10−12^ Finally, nonthermal plasmas produce highly pure spherical Si particles of controllable size, but these particles are then collected as a powder or loosely packed on a substrate, rather than as a dispersion in solution ready for further processing.^13,14^ Thus, we still aspire toward a room-temperature, solution-chemistry based method, a cousin to the Stöber method for silica, producing crystalline silicon particles.

Ligand-controlled growth, a popular technique used in other metallic and metal oxide particle syntheses, is less explored in the growth toward large Si particles. Ligand-controlled growth typically involves aqueous solvents and oxygen-containing precursors, which cannot be transferred to silicon nanochemistry due to the spontaneity of silicon to oxidize or hydrolyze in the presence of oxygen or hydroxyl groups. Seemingly transferrable, however, is the preparation of nanoparticles from organometallic precursors in organic solvents. Organometallic precursors provide dual functionality: (1) they can be decomposed under mild conditions, in comparison to traditional precursors with high Si bond strengths, and (2) upon decomposition they can provide ligands that stabilize the particle surface.

Amidinate ligands are known to form stable complexes with most transition-metals and, due to their ease of decomposition, have been explored as metallic nanomaterial precursors.^15,16^ Amidinate-based coordination complexes have yielded amidinate surface-stabilized metallic nanoparticles.^17^

While Si amidinate complexes have been synthesized with a variety of ligand-alterations,^18^ only recently have we used this type of complex successfully in particle synthesis,^19^ through the redox reaction between a silicon Zintl phase and an amidinate-stabilized silicon(IV) precursor. In our previous study, a stoichiometric 1:1 ratio between the precursors generated particle sizes from 15 to 45 nm, where particle size was altered by changing the dispersive media. Here, we chose the solvent producing the largest particles (toluene) and tuned the stoichiometric ratio Na_4_Si_4_/SiCl_2_[BuC(N^i^Pr)_2_]_2_ between 1 and 4, to control particle size from 45 to 230 nm. Individual particle refractive index measurements were made on the largest particles produced (*d* ≈ 230 nm) and the refractive index was found to be near that of bulk crystalline Si.

## Results and Discussion

**Scheme 1:**
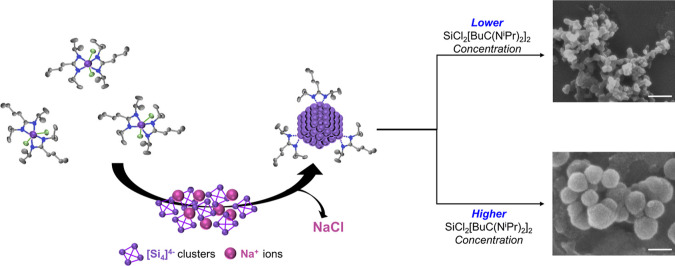
Reaction Mechanism of SiCl_2_[BuC(NiPr)_2_]_2_ toward Si Particles Purple indicates Si, green Cl, blue N, and grey C. Scale bars represent 200 nm.

This interfacial redox reaction occurs at room temperature, between a solution phase silicon coordination complex, bis(*N*,*N*′-diisopropylbutylamidinato)dichlorosilane, and a solid phase Zintl salt, sodium silicide, which is synthesized to a purity of ca. 98 mol %.^20^ The structure of SiCl_2_[BuC(N^i^Pr)_2_]_2_ is shown in Figure S1. The initial reaction between anionic [Si_4_]^4–^ subunits and Si^4+^ species is most likely rapid, due to the large difference in redox potential between the two precursors. The molar ratio of the solid and dissolved silicon precursors affects the final particle diameter, as shown in Scheme 1.

**Figure 1:**
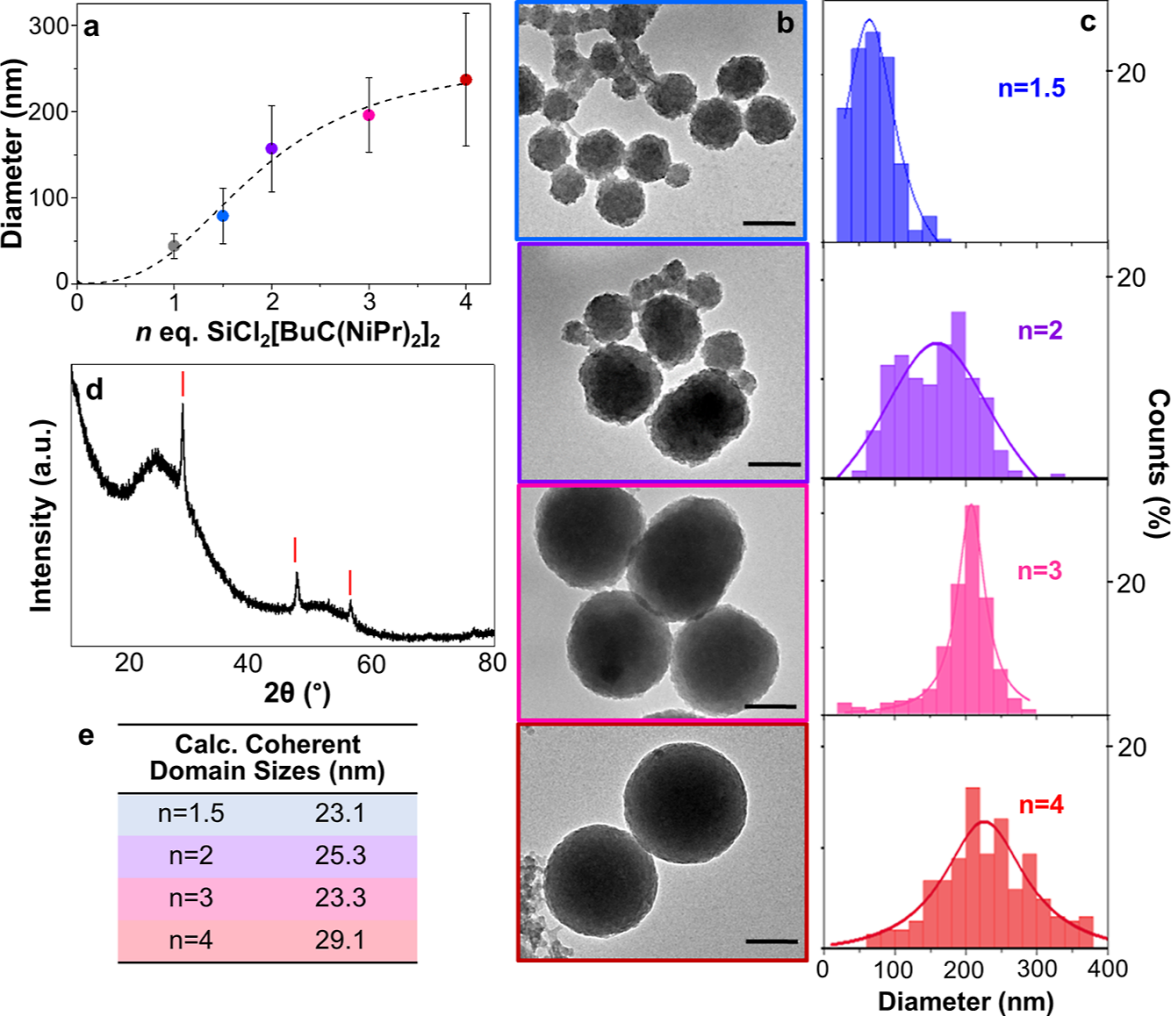
(a) Median particle size vs *n* SiCl_2_[BuC(N^i^Pr)_2_]_2_ molar equivalents plotted and fitted with a logistic trend line. Vertical bars show the standard deviation of each batch. (b) TEM images for varying precursor ratios with *n* = 1.5 to 4. All scale bars represent 100 nm. (c) Corresponding particle size distributions from counting 200 particles. (d) Typical powder X-ray diffractogram obtained from Si particles with *n* = 2. (e) Calculated coherent domain sizes.

First, we tested the reaction using an excess of the reducing agent, sodium silicide (1.5 Na_4_Si_4_/1 SiCl_2_[BuC(N^i^Pr)_2_]_2_). An excess of sodium silicide forms small, highly aggregated silicon particles (Figure S2) of 20–40 nm. Nonaggregated silicon nanoparticles in this size range have already been produced using commercially available silanes,^21−24^ and thus were not characterized any further. More interesting was what happened to particle size when we used an excess molar ratio of SiCl_2_[BuC(N^i^Pr)_2_]_2_ with respect to sodium silicide. When 1 Na_4_Si_4_ to 1.5 SiCl_2_[BuC(N^i^Pr)_2_]_2_ were reacted, particle size nearly doubled. Several additional molar ratios (*n*) were tested and particle sizes continually increased, yielding average diameters between 45 and 230 nm, when using between *n* = 1–4 equiv of SiCl_2_[BuC(N^i^Pr)_2_]_2_ (Figure 1a,b). The error bars are related to the polydispersity within a batch. Particle size distributions are shown in Figure 1c (nonspherical objects were not counted in the shown size distributions). Above *n* = 4, the particles became even more irregular in shape (Figure S3). For the current synthesis conditions, the sizes attainable are well-defined by the parameters shown in Figure 1a. By simply adjusting the molar ratio of the two silicon precursors, particle sizes that display Mie resonance across the visible spectrum can be obtained.

A typical X-ray powder diffractogram of the obtained particles confirms the presence of crystalline Si (Figure 1d), where the broad contribution around 2θ = 25° may indicate the presence of amorphous silicon or partially oxidized particles. Coherent domain sizes were calculated for each batch of particle sizes (Figures 1e, S4) and remain relatively similar at about 25 nm, irrespective of the particle diameter derived from TEM. This may suggest that the initial size of nucleated particles, or germs, is relatively constant, and particle growth may occur through aggregation of primary particles.

We found that the reaction proceeds without needing to first isolate the molecular complex. This prompted us to investigate whether the effect of additional Si(IV), ligand or both contribute to the growth of Si particles. The quantities of amidinate ligand and silane, in the form of SiCl_4_, were independently varied relative to a fixed quantity of sodium silicide (Figure S5). First, let us specify that these reactions differ from previous reactions performed using SiCl_4_,^21,22,24^ because the silane reacts with the lithium amidinate ligand in situ, prior to the reaction with sodium silicide, allowing the reaction to occur at room temperature (whereas the other studies took place at 85 °C). Notably, both the amidinate and the Si(IV) concentration affect the final particle size and shape. Increasing the concentration of Si(IV) increases particle size when the amount of ligand is held constant. When both concentrations are low, particles are small and highly agglomerated. Larger and more separated particles are obtained when multiple equivalents of both ligand and silane are used, relative to sodium silicide. Finally, we found that the order of addition is crucial, since when the ligand is not preassociated with the silane (that is added after mixing Zintl salt and silicon tetrachloride), the resulting particles are more polydisperse. This indicates that forming the complex prior to contact with the sodium silicide yields silicon batches of better quality.

Increased particle size with excess ligand is seemingly contrary to typical ligand-controlled nanoparticle growth, where higher ligand concentrations in solution typically yield smaller nanoparticles.^17,25^ However, higher ligand concentrations do not give smaller particle sizes when the growth mechanism proceeds via aggregation and coalescence. Here, the particles are formed in a nonpolar solvent, toluene. There are no strong repulsive forces due to a highly polarizable solvent, electrostatic surface charges, or steric forces from large surface-bound polymers. Thus, van der Waals attractive forces can lead to a certain degree of aggregation.^26^ This is consistent with TEM images showing aggregated objects. We also probed particle growth as a function of time and stopped the reaction after 1 and 4 h (Figure S6). Smaller particles were observed by TEM, and although the sample is too aggregated to construct a size distribution, the average particle size appears to increase with time, reaching a maximum at 16 h. In order to better understand the system, the surface chemistry requires analysis.

**Figure 2:**
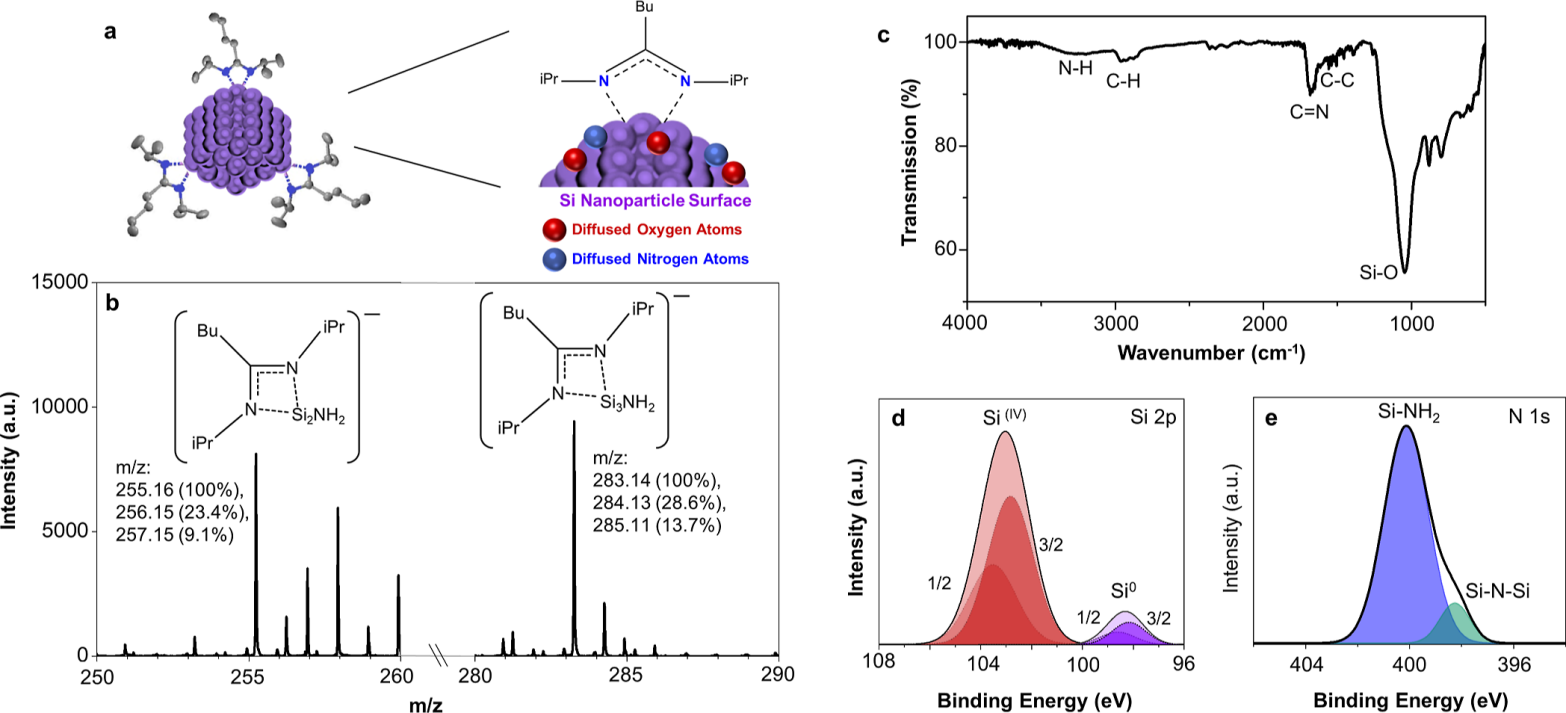
(a) Schematic showing particle surface chemistry where Si atoms are indicated in purple, oxygen in red, nitrogen in blue and carbon in gray. (b) ToF-SIMS mass spectra (negative mode) subset showing major peaks obtained from particles. (c) ATR-FTIR of solid particle film. High resolution XP spectra of (d) Si 2p region (black line corresponds to fit data. 2p_3/2_ and 2p_1/2_ peak fittings are shown). High resolution XP spectra of (e) N 1s region (black line corresponds to the fit data). Data corresponds in all cases to particles formed using *n* = 2 equiv SiCl_2_[BuC(N^i^Pr)_2_]_2_.

Negative ToF-SIMS ion spectra indicate the presence of silicon clusters coordinated to amidinate ligands with intense peaks at *m*/*z* 255.2 and 283.1 (Figure 2b). Amine ions are also attached to these clusters. The fragments associated with each of these peaks, separated by the mass of one Si atom, are shown above each peak. The isotopic distribution also corresponds well with these fragments, as observed in subsequent peaks. Ion mapping is provided in Figure S7 to show the spatial distribution of the ions concentrated in regions containing the particles. For comparison, ion mapping for Au^–^ is shown, ensuring that the amidinate ligand comes directly from the particles and not from some residue on the gold substrate. The residual nitrogen present on the surface of the particles is likely due to reaction with the solvent used to wash the silicon particles. It has been previously shown that dimethylformamide reacts with a silicon surface.^26^ It is thus likely that formamide is the source of both the oxygen and the amine groups found on the particle surface.

X-ray photoelectron spectroscopy (XPS) and ATR-FTIR spectroscopy support the ToF-SIMs interpretation (Figure 2c–e). A strong vibrational mode for SiO_*x*_ is centered at 1040 cm^–1^ and a weaker mode around 880 cm^–1^.^27^ A vibrational mode emerges at approximately 1680 cm^–1^ which can be assigned to the amidinate ligand C=N stretching. Alkyl C–H vibrational modes are seen just below 3000 cm^–1^ and a weak, broad mode at 3300 cm^–1^ indicates the presence of primary amine. These two peaks are present for all particle sizes with no significant difference in peak height ratio from one sample to the next (Figure S8). XPS results are also consistent with the presence of amidinate ligand and primary amine on the surface (Figure 2d,e). Enlargement of the Si 2p region shows two distinctive peaks corresponding to Si(0) core atoms with a peak at 98.3 eV and surface Si(IV) atoms attached to either N or O, with a peak at 103.0 eV. The N 1s region can be fit to two components corresponding to Si–N–Si (398.3 eV) and primary amine (400.1 eV).^28^ Thus, ATR-FTIR spectroscopy and XPS indicate that the surface contains oxygen, amine and amidinate functionalities.

**Figure 3:**
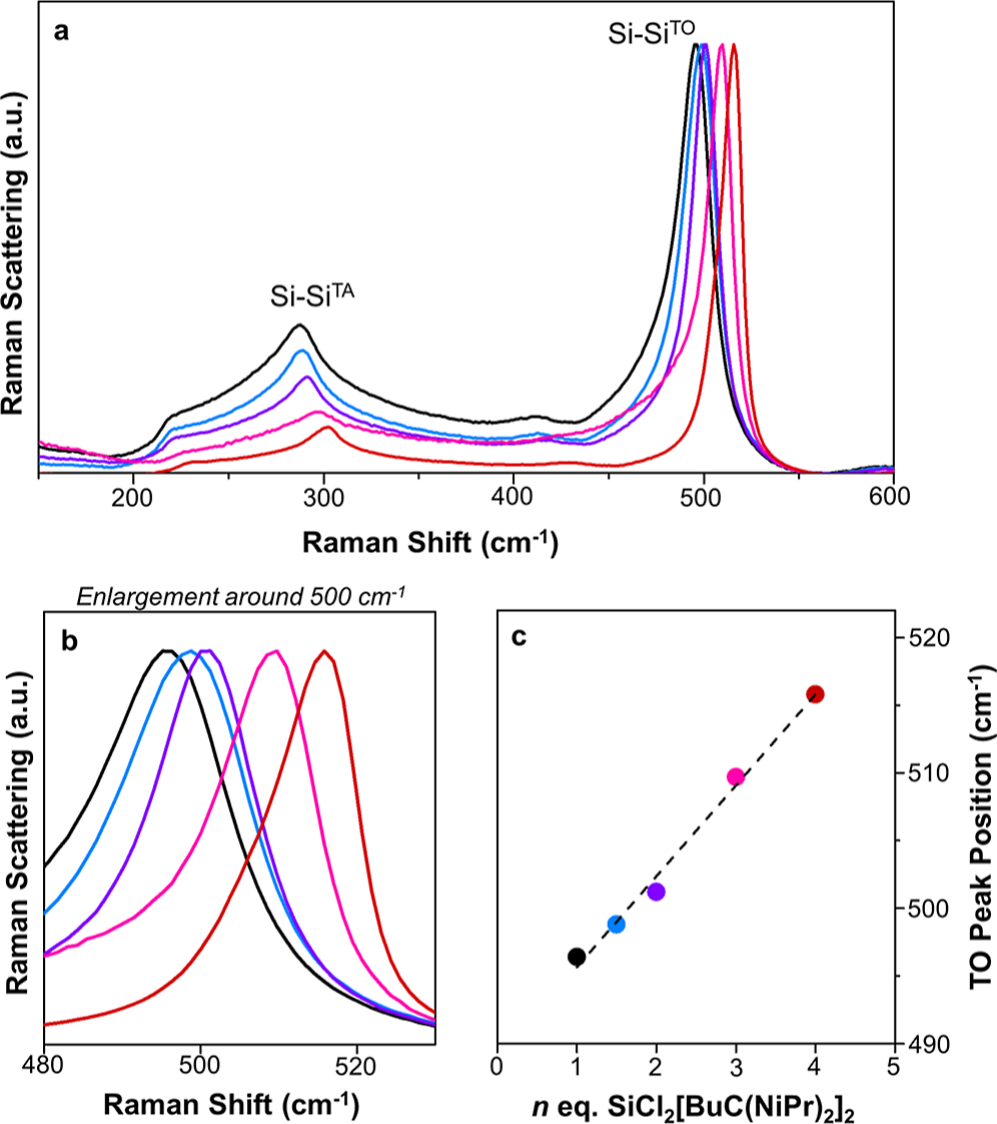
(a) Raman spectra of particles produced using 1 to *n* Na_4_Si_4_ to SiCl_2_[BuC(N^i^Pr)_2_]_2_ in toluene at room temperature. (black) 1 equiv, (blue) 1.5 equiv, (purple) 2 equiv, (pink) 3 equiv, and (red) 4 equiv [BuC(N^i^Pr)_2_]_2_SiCl_2_. (b) Enlargement of the region of the Si–Si TO vibrational mode, (c) maximum Raman peak shift for each batch of particles.

The particles were characterized by Raman spectroscopy to probe the silicon environment more fully (Figure 3). The two main peaks related to the light scattering of the transverse optical (TO) and transverse acoustical (TA) photons are observed for all of the samples. The TO-related peak is broad and located at 480 cm^–1^ for an amorphous Si film, and at 520 cm^–1^ for bulk crystalline Si.^28^ For the largest particles (∼230 nm) the TO band is at 515 cm^–1^ and the TA band is at 300 cm^–1^, both of which are close to that of bulk crystalline Si. When the size of the Si particles decreases, both bands shift to the red (i.e., to lower Raman shifts) and become more dissymmetric. Band shifting and broadening has been associated with the decrease of nanoparticle sizes due to quantum confinement effects,^29−32^ however the particles made here are probably too large to display sufficient quantum confinement. Another potential explication is the amorphization of the silicon particles, however the line shape does not really correspond to a progressive amorphization.^33^ The red shift and the band asymmetry of TO Raman peaks could potentially originate from a Fano interference of the TO phonon with an electronic continuum, as reported for smaller Si nanoparticles.^34^ However, the red shift, which is accompanied by a decrease in the intensity of the TO band, could also be related to a partial oxidation.^35,36^ Greater oxidation for smaller particles can be explained by their greater surface area to volume ratio.

**Figure 4:**
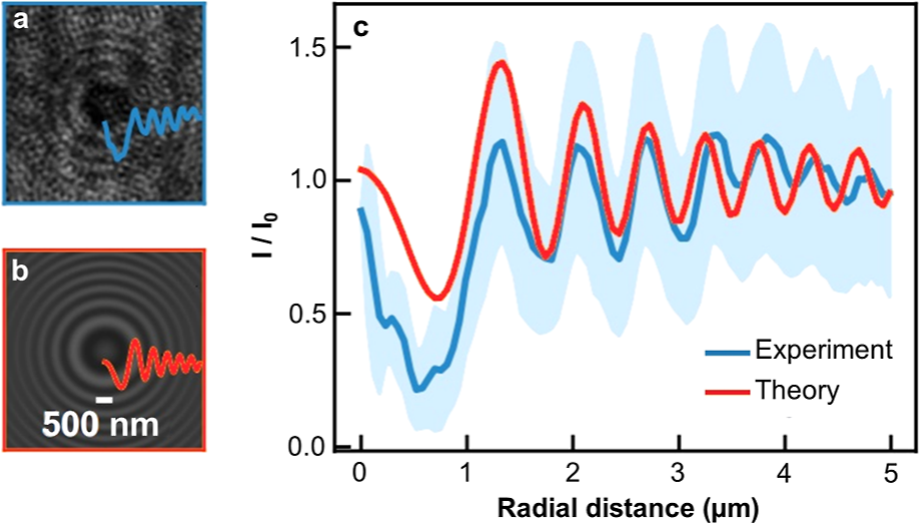
(a) Experimental interference pattern captured by a single particle produced from particles formed using *n* = 4 equiv SiCl_2_[BuC(N^i^Pr)_2_]_2_. (b) Corresponding best-fit Lorenz–Mie interference pattern, providing the radius *a* = 113 ± 10 nm and refractive index *n* = 4.08 ± 0.03 of the particle. (c) Angular averages of the intensities *I* (normalized by the mean intensity *I*_0_) from the experimental and theoretical interference patterns, as functions of the radial distance to the *z*-axis.

The refractive index was measured of an individual particle with a median diameter of 230 nm (Figure 4). The optical microscope measuring the refractive index has a resolution limit near that of our largest batch of particles (*n* = 4 equiv SiCl_2_[BuC(N^i^Pr)_2_]_2_), thus only the refractive index of our largest particle sample could be determined. With a 532 nm laser, the refractive index for a 113 ± 10 nm radius particle was found to be 4.08 ± 0.03 when the inference pattern from a single particle was fit with a Lorentz–Mie model via a previously established protocol.^37,38^ The distribution of measured indices of 170 images is shown in Figure S9. For comparison, in bulk crystalline Si, at this wavelength, the refractive index is 4.14. The measured refractive index of these large Si particles corresponds well to the highly crystalline nature observed with Raman spectroscopy for the largest particle batch.

## Conclusion

The control of particle sizes, in the size ranges of hundreds of nm, is unprecedented for solution syntheses of silicon particles. In the interfacial reaction reported here, particles with a tunable diameter between 45 and 230 nm can be produced by using an excess of silicon complex, SiCl_2_[BuC(N^i^Pr)_2_]_2_, with respect to the Zintl phase, Na_4_Si_4_. The systematic variation of SiCl_2_[BuC(N^i^Pr)_2_]_2_ in solution resulted in a positive correlation between precursor ratio and particle size. The precursor was deconstructed into its Si(IV) and ligand components, which also produced Si particles with an increase in particle size observed for both greater Si(IV) and ligand quantities. Thus, this colloidal synthesis route opens future exploration of other ligands for the size and shape control of Si particles. Increases in ligand concentration in solution did not impact particle size in the expected manner: more ligand produced larger particles. The surface binding of amidinate ligands on the surface of the particles is confirmed by ToF-SIMS, ATR-FTIR, and XPS. Optical refractive index near that of bulk silicon has been observed, showing promise for these nano-objects in optically responsive materials, although the polydispersity should be improved upon in future development. Particles of tunable size and form are equally needed in the anodes of lithium ion batteries.

## Experimental Methods

### Materials

Toluene (anhydrous, 99.8%), *N*,*N*′-diisopropylcarbodiimide (99%), silicon tetrachloride (99%), *n*-butyllithium (2.5 M in hexane), and NaH (90%) were purchased from Sigma-Aldrich and used without further purification. Silicon particles were purchased from Nanomakers (40 nm). Tetrahydrofuran (THF, 99.9%) was obtained from Sigma-Aldrich and purified in an MBraun MBSPS 5 solvent purification system. Formamide (99%) was purchased from Fischer Scientific and the oxygen was removed by three freeze–pump–thaw cycles. The purified solvent was stored over 3 Å molecular sieves in an argon filled glovebox. Carbon-coated copper grids were purchased from Electron Microscopy Sciences (Hatfield, PA). Miniature hollow glass capillaries were purchased from CMScientific (path length 0.2 mm, width 2 mm and 0.14 mm). All the experiments were carried out under inert atmosphere, with Schlenk line techniques, or in an argon-filled glovebox.

### Synthesis of Bis(*N*,*N*′-diisopropylbutylamidinate)dichlorosilane (SiCl_2_[BuC(N^i^Pr)_2_]_2_)

The silicon precursor was prepared according to a previously reported protocol.^19^ First, butyllithium (13 mL, 0.032 mol) was added to a solution of *N*,*N*′-diisopropylcarbodiimide (5.0 mL, 0.032 mol) in THF (80 mL) in a cooling bath slurry consisting of a 60/40 v/v % ethanol/water mixture with liquid N_2_ (−40 °C). The solution was allowed to come to room temperature, and stirred overnight. This reaction yields an intermediate, lithium amidinate. In a second step, SiCl_4_ (1.86 mL, 0.016 mol) was added in a cooling bath slurry consisting of a 70/30 v/v % ethanol/water mixture (−80 °C) with liquid N_2_. The solution was allowed to come to room temperature, and stirred overnight. The THF was evaporated and substituted with toluene (60 mL). LiCl precipitated and was filtered out of the media via cannula filtration. Most of the toluene (50 mL) was removed via evaporation to concentrate the product. The product was stored at −30 °C until crystals formed. Then, cold toluene (30 mL) was added and the product was further chilled at −30 °C overnight. The next day, larger, colorless crystals had formed. These were collected and washed with cold toluene. The precursor was stored at −30 °C.

### Synthesis of Sodium Silicide, Na_4_Si_4_

High purity Na_4_Si_4_ was prepared according to a previously published protocol.^20^ In a typical batch, NaH (19.6 mmol, 470 mg) and silicon nanoparticles (17.9 mmol, 500 mg) were mixed together at 20 Hz for 2 min using a ball mill (Retsch MM400 ball miller, airtight vials of 50 mL, one steel ball of 62.3 g, and a diameter of 23 mm). The powder was recovered in an argon filled glovebox and put into a h-BN crucible, which was placed in an airtight quartz tube. The quartz tube was placed inside a vertical oven and connected to an Ar flow of 0.06 L min^–1^. A heating ramp of 10 °C min^–1^ was used to 400 °C, followed by a dwell time of 24 h. Finally, the sample was cooled naturally. The reaction vessel was then transferred back to an argon-filled glovebox and the synthesized powder was recovered and stored in the glovebox.

### Reactions of SiCl_2_[BuC(N^i^Pr)_2_]_2_ with Na_4_Si_4_

In an argon-filled glovebox, Na_4_Si_4_ was weighed and added to a round-bottom Schlenk flask equipped with a magnetic stir bar. Toluene (8 mL) was added, followed by the appropriate molar eq of SiCl_2_[BuC(N^i^Pr)_2_]_2_ (Table S1), maintaining a silicon concentration of 20 mM. The reagents were stirred with magnetic agitation at room temperature for 16 h under a flow of argon gas. After the synthesis, the products were collected under inert atmosphere. The crude product, appearing as a black solid, was washed twice with anhydrous formamide, and twice with anhydrous THF via 10 min centrifugation at 10 400*g*.

### Reactions of SiCl_4_, with or without Lithium Amidinate and Na_4_Si_4_

In an argon-filled glovebox, 4 mg Na_4_Si_4_ was weighed and added to a round-bottom Schlenk flask equipped with a magnetic stir bar. Toluene (4 mL) was added, followed by the appropriate molar eq of SiCl_4_ and/or lithium amidinate corresponding to Figure S6. The reagents were stirred with magnetic agitation at room temperature for 16 h under a flow of argon gas. After the synthesis, the products were collected under inert atmosphere and washed with the same protocol mentioned above.

### Transmission Electron Microscopy

Particles were dispersed in THF and then sonicated for 3 min. One drop was cast onto a copper grid covered with a carbon film (previously activated by UV, carbon film, face up) for analysis by TEM. Bright field TEM images were acquired using a JEOL 1400+ (JEOL, Tokyo, Japan) microscope operating at an acceleration voltage of 120 kV and equipped with a Smart Orius 1000 camera obtained from GATAN.

### Scanning Electron Microscopy

Samples were drop cast onto aluminum sample holders and SEM images were acquired using a field emission scanning microscope (JSM 6700F, JEOL 6700F) microscope operating at a current of 10 mA.

### Powder X-ray Diffraction

X-ray diffractograms from 8 to 80° 2θ were recorded on dried, ground samples spread evenly onto a zero-background sample holder using a PANalytical X’Pert Pro Apparatus (Cu radiation source λ = 0.15418 nm) equipped with an X’Celerator detector.

### Time-Of-Flight Secondary Ion Mass Spectrometry

Samples were drop cast onto gold-coated substrates and experiments were performed using a TOF.SIMS 5 spectrometer (IONTOF GmbH) equipped with a liquid metal ion gun oriented at 45° to the sample. The diameter of the 30 kV Bi^3+^ ion beam was approximatively 5 μm. The beam was operated at a 0.3 pA ion current in spectrometry mode and raster scanned over the surface to generate 500 × 500 μm secondary ion images.

### Infrared Spectroscopy (ATR-FTIR)

Measurements were recorded on a solid film on a Shimadzu IRAffinity-1S Fourier Transform Infrared (FTIR) spectrometer with a MIRacle 10 Single attenuated total reflection Accessory.

### X-ray Photoelectron Spectroscopy

Powdered samples were pressed onto indium strips and XPS was carried out with a Thermo Scientific Kα with an Al Kα X-ray source and a spot size of 400 μm. Quantification and peak fitting were achieved by using the AVANTAGE software (Thermo Fisher Scientific).

### Raman Spectroscopy

Samples were prepared by drop casting particles dispersed in THF on a microscope slide in the glovebox. Raman spectra were recorded on an Xplora spectrometer (Horiba), equipped with a confocal microscope. An objective lens with a 10× magnification was used to observe the sample. An Olympus LM Plan FLN 100× objective lens with 0.80 numerical aperture and 3.4 mm working distance was employed to focus the laser beam on the sample and collect the scattered light. A long-pass edge filter was used to remove the fundamental line from the collected scattered light. The spectra were run using a 632.81 nm HeNe gas laser, using a filter to reduce the laser power to 20% of the total power. A 600 lines per mm grating was used to separate the scattered light into its components that were collected onto a Syncerity TE-cooled FI–UV–vis detector. Three measurements in three different zones were collected for each sample, to assess the reproducibility of the measurement and the uniformity of the sample.

### Refractive Index Measurement

A dilute suspension of particles dispersed in anhydrous THF was drop casted on a microscope slide within a vacuum grease ring. A coverslip was placed over the droplet of particles, which prevented the volatile solvent from evaporating. The refractive index of an isolated particle is measured by in-line Mie holography.^38^ A plane wave (wavelength: 532 nm) illuminated the particle. The light scattered by the particle interfered with the incident beam onto the focal plane of an oil-immersed Olympus objective lens (× 100, NA 1.45). The resulting 2-dimensional pattern (Figure 4a) was then fit to the Lorenz–Mie theory, as detailed in ref (37). The obtained theoretical pattern (Figure 4b) captures the experimental pattern, as evidenced by the intensity profiles of the patterns, thus allowing for precise measurement of particle size and refractive index.

## Data Availability

Data for this article, including median particle size quantification, TEM bright field images, powder diffractograms, single attenuated total reflection Fourier transform infrared spectrometry, X-ray photoelectron spectra and Raman spectra are available at Zenodo at 10.5281/zenodo.12943336.

## Supporting Information Available

Molecular structure of bis(*N*,*N*′-diisopropylbutylamidinate)dichlorosilane, TEM images of particles produced when sodium silicide is reacted with SiCl_2_[BuC(NiPr)_2_]_2_ in toluene at 1.5:1 and 1:5 ratios, powder X-ray diffractograms of all samples, TEM images from additional experiments varying the quantity of Si(IV) species and ligand separately to a fixed quantity of Na_4_Si_4_, TEM images from growth experiments stopped early, ToF-SIMS images, ATR-FTIR spectra of all samples, additional experimental details, distribution of measured indices computed from 170 interference pattern images (PDF)
