# Supplementary material for: Unveiling the Potential of Redox Chemistry to Form Size-Tunable, High-Index Silicon Particles
Source: Chem Mater. 2024 Aug 28;36(22):10986–93. doi: 10.1021/acs.chemmater.4c01439 (PMC11603531; doi:10.1021/acs.chemmater.4c01439)
Supplement: Supplementary file 1 — cm4c01439_si_001.pdf [file cm4c01439_si_001.pdf]

Supporting Information

Unveiling the potential of redox chemistry to form size tunable, high index silicon particles

Megan A. Parker[a], Safa Khaddad[a], Nicolas Fares[b], Anissa Ghoridi[c], David Portehault[c], Sébastien Bonhommeau[d], Yacine Amarouchene[b], Patrick Rosa[a,*], Mathieu Gonidec[a,*], Glenna L. Drisko[a,*]

[a]Univ. Bordeaux, CNRS, Bordeaux-INP, ICMCB, UMR 5026, F-33600, Pessac, Fr

[b]Univ. Bordeaux, CNRS, LOMA, UMR 5798, F-33405, Talence, Fr

[c]Sorbonne Université, CNRS, LCMCP, F-75005 Paris, Fr

[d]Univ. Bordeaux, CNRS, Bordeaux-INP, ISM, UMR 5255, F-33400, Talence, Fr

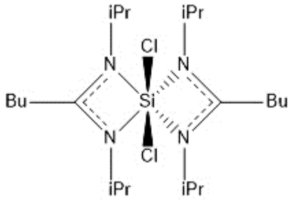

Figure S1. Molecular structure of bis(N,N'-diisopropylbutylamidinate) dichlorosilane. Referred to as $SiCl_2[BuC(N^iPr)_2]_2$ throughout this publication.

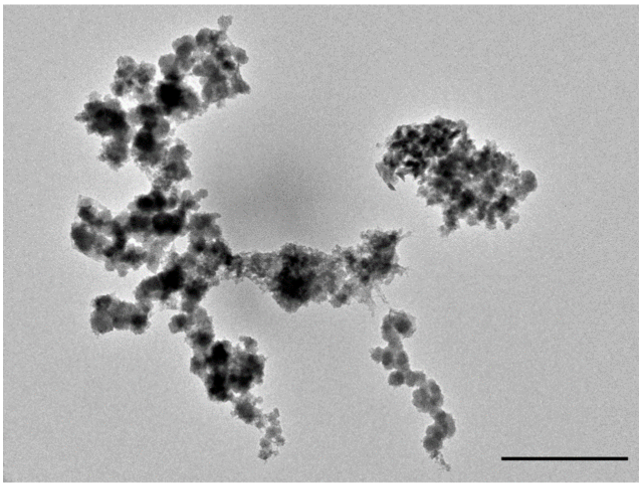

Figure S2. Particles produced when excess sodium silicide is reacted with $SiCl_2[BuC(N^iPr)_2]_2$ in toluene (1.5 : 1 molar ratio). Scale bar represents 1 µm.

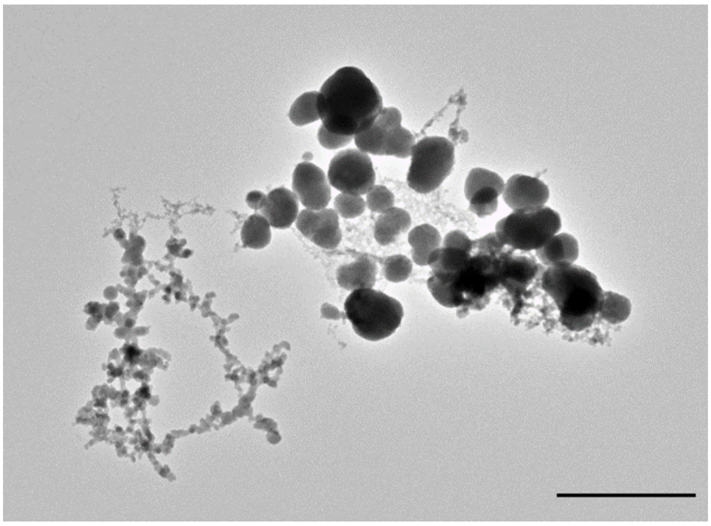

Figure S3. TEM image of particles formed when 5 molar eq. $SiCl_2[BuC(N^iPr)_2]_2$ is reacted with 1 molar eq. Na_4Si_4 in toluene for 16 hours. Scale bar represents 1 µm.

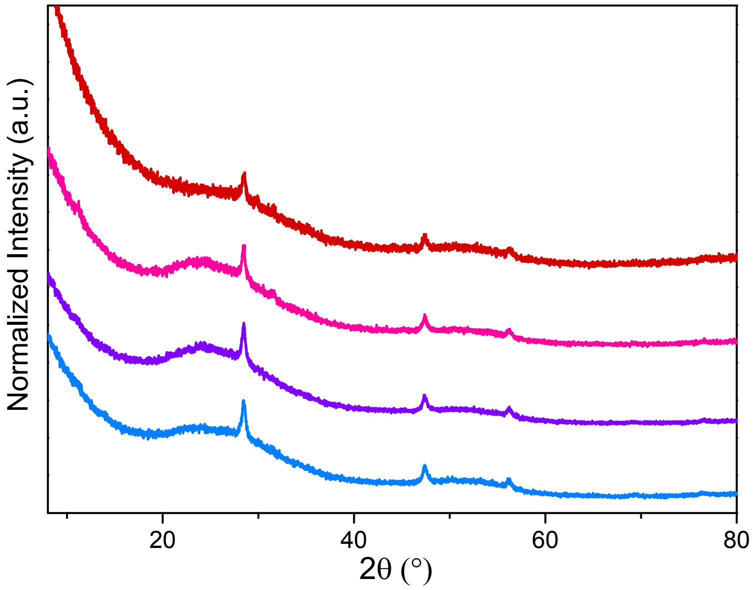

Figure S4. Powder X-ray diffractograms of particles produced when sodium silicide is reacted with $SiCl_2[BuC(N^iPr)_2]_2$ in toluene for 16 hours with a (blue) 1:1:5 molar ratio, (purple) 1:2 ratio, (pink) 1:3 ratio, and (red) 1:4 ratio.

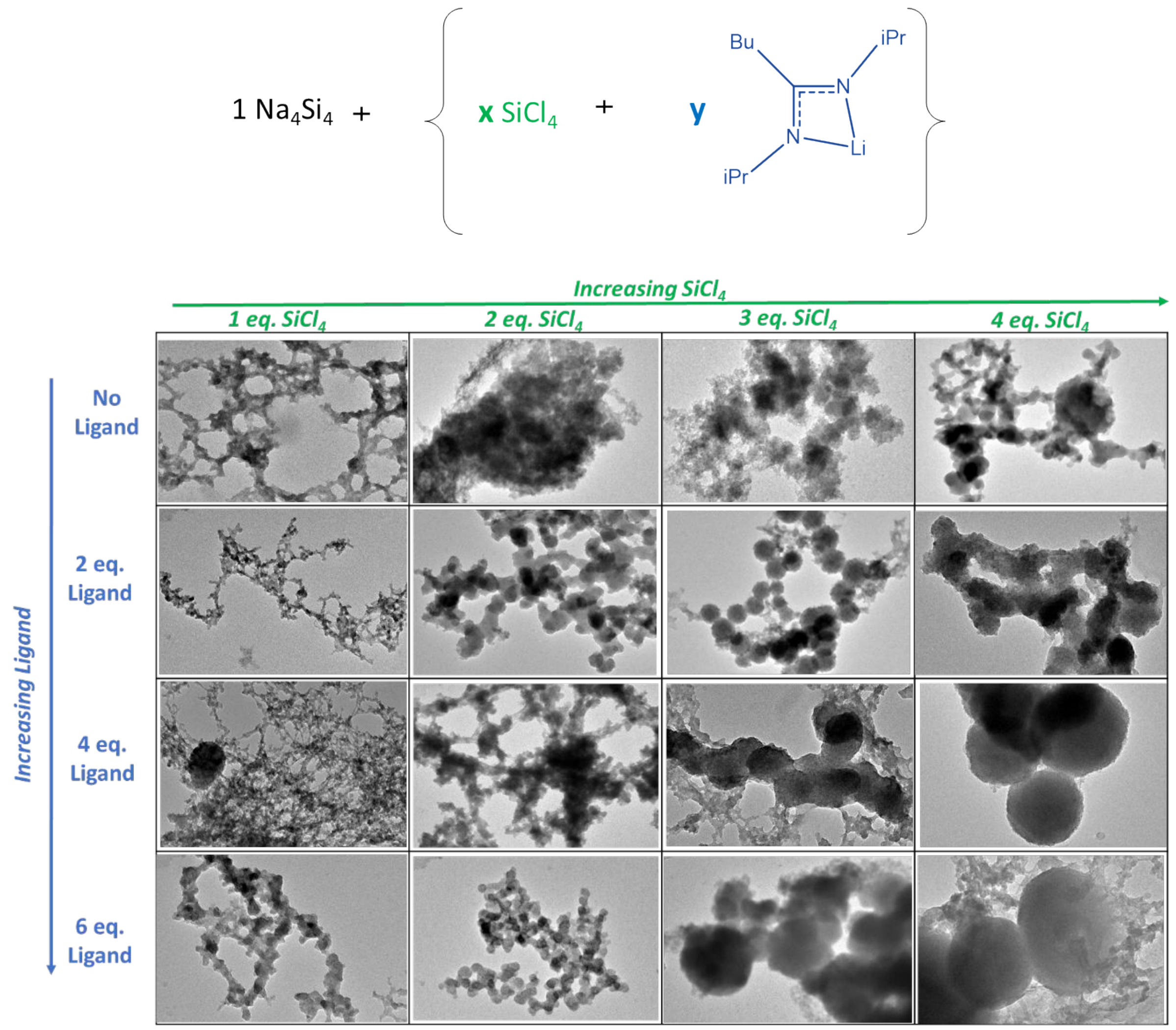

Figure S5. Additional experiments varying the quantity of Si (IV) species and ligand separately to a fixed quantity of Na_4Si_4 in toluene and reacted for 16 hours. An overall reaction concentration of 20 mmol was maintained in all cases.

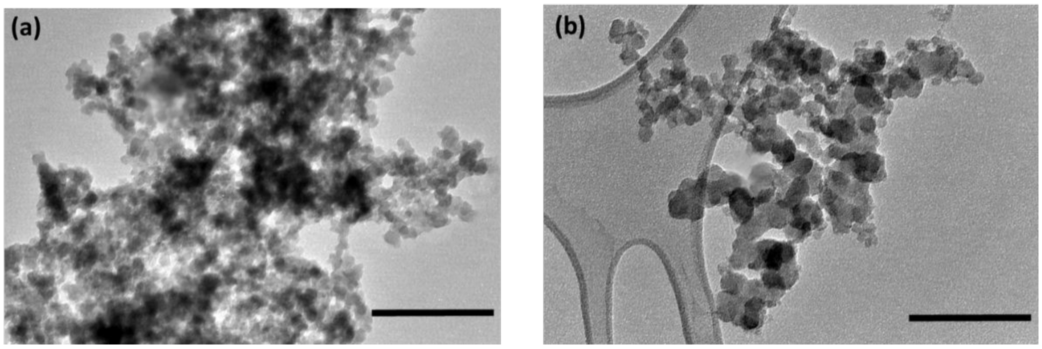

Figure S6. Growth experiments stopped early, (a) after 1 hour and (b) after 4 hours using 2 eq $SiCl_2[BuC(N^iPr)_2]_2$ with 1 eq. Na_4Si_4 in toluene. Scale bars represent 200 nm.

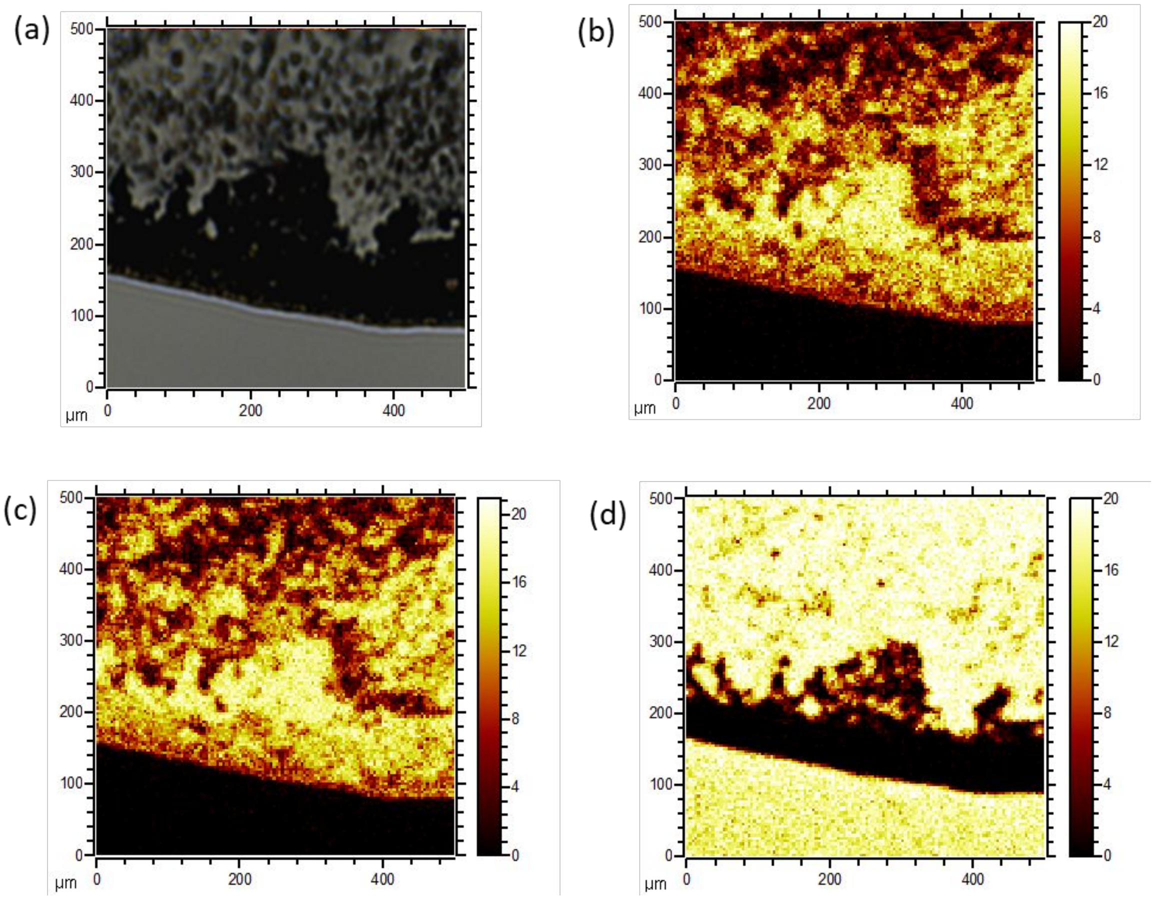

Figure S7. Optical image (a) and ToF-SIMS images of particles formed using n=2 eq. $SiCl_2[BuC(N^iPr)_2]_2$ corresponding to (b) m/z peak at 255, (c) m/z peak at 283, (d) m/z peak for Au^-.

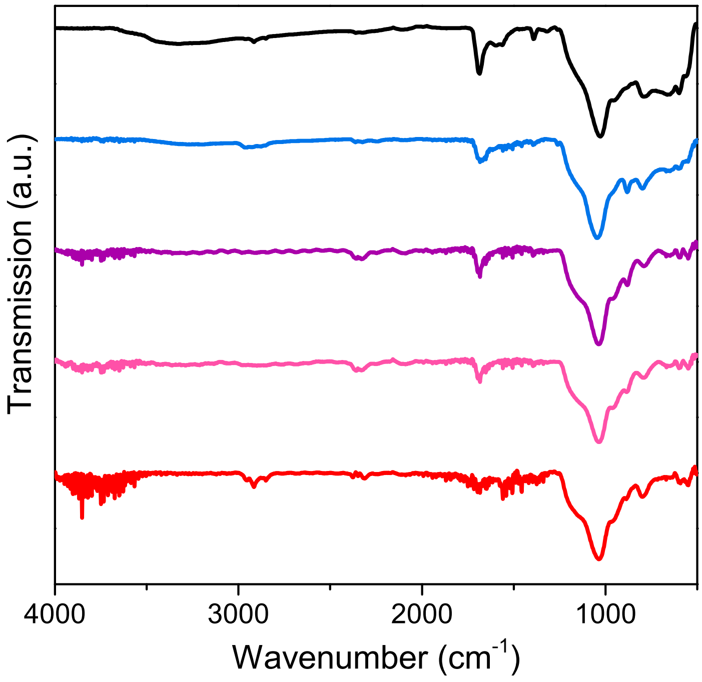

Figure S8. ATR-FTIR spectroscopy of particles produced when sodium silicide is reacted with $SiCl_2[BuC(N^iPr)_2]_2$ in toluene for 16 hours with a (black) 1:1, (blue) 1:1:5, (purple) 1:2, (pink) 1:3, and (red) 1:4 molar ratio.

Table S1. Actual precursor quantities used to maintain 20 mmol concentration in 8 mL toluene.

Molar Ratio	mg $SiCl_2[BuC(N^iPr)_2]_2$	mg Na_4Si_4
1 : 1	37.4	16.5
1 : 1.5	44.3	13.2
1 : 2	49.9	11.0
1 : 3	54.5	9.0
1 : 4	59.9	6.6
1 : 5	62.4	5.5

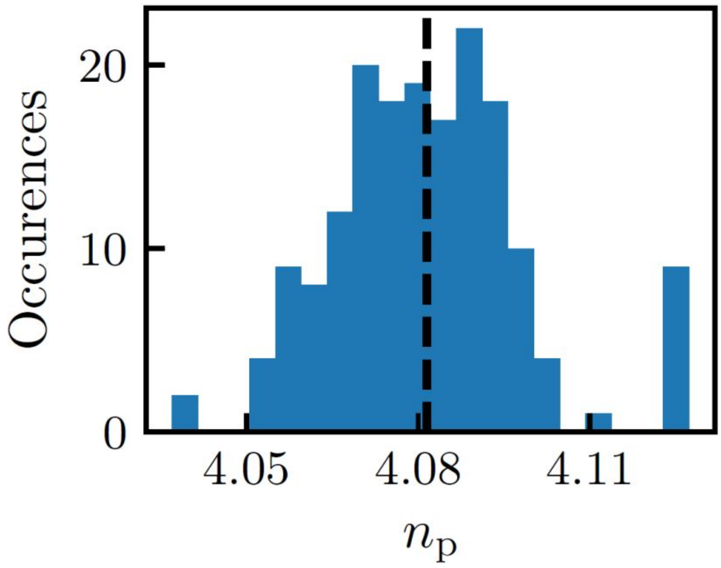

Figure S9. The distribution of measured indices computed from 170 interference pattern images, captured by a single particle formed using n=4 eq. $SiCl_2[BuC(N^iPr)_2]_2$.
